# Bargaining for the Supply of Medicines

**Published:** 1905-11-18

**Authors:** 


### Bargaining for the Supply of Medicines.

A case was heard recently in the King's Bench Division of the High Court before Mr. Justice Wills and Mr. Justice Darling, and on appeal from the Brompton County Court, which very well illustrates some of the disadvantages which may arise from bargains between medical practitioners and druggists for the supply of medicines to the patients of the former; not to say from every form of introducing the commercial, as distinguished from the professional, element into the relations between the sick man and the physician. The report of the case which we have seen was rendered less lucid than was desirable by uncertainties connected with the eccentric employment of a personal pronoun, uncertainties calculated to remind a Devonian of a well-known local narrative commencing "He'd a stick and he'd a stick and he hit he and he hit he and if he, etc., etc." Making allowance for these uncertainties, the facts appear to be that the plaintiff, Mr. Adams, a qualified medical practitioner, was employed by Mr. Butterworth, the defendant, to attend his mother. An agreement was made as to charges for attendance, but nothing was said as to medicine. The plaintiff duly attended, and, having paid his visit, he "took away a prescription (presumably of his own writing) and had it made up," not by the defendant's customary druggist, but by one of his own selection, who lived a mile and a half from the defendant's house, and with whom the plaintiff appears to have had a contract for the supply of medicines to his patients, a contract on which his own charges for them represented a profit of 50 per cent. The defendant objected to the distance of the new druggist, and the plaintiff is said to have explained that he made the change in order that he might be able to control the supply of medicine to the patient. This, according to the evidence, he failed to do; for whereas he had prescribed for her two doses of chloral a day, she had sent for and had taken three doses a day, obtaining them from the plaintiff's druggist. These additional doses were admitted by the plaintiff to have been taken without his knowledge and to have been harmful to the patient. He ultimately sent in an account charging £34 5s. 6d. for attendance and £8 17s. 2d. for medicines, a sum which included the extra doses of chloral, and represented the aforesaid profit of 50 per cent. upon the charges made to him by the druggist. The defendant repudiated some of the particulars; and, on being sued in the Brompton County Court, paid into court £29 18s. for medical attendance, and £5 16s. 8d. for medicine, amounts which were found by the County Court Judge, after hearing evidence, to be sufficient. Against this judgment the plaintiff appealed, and his appeal was dismissed by the High Court. Concerning the defendant's reasons for disputing part of the charge for attendance the report is silent, but with regard to the medicines we find it difficult to regret the plaintiff's failure to convince the judges. A medical practitioner who dispenses his own medicine may reasonably ask for a profit upon it, as a recompense for the time and skill occupied in compounding and for the capital embarked in keeping the necessary stock of drugs in good condition. But a medical practitioner who avoids these expenses, who demands proper fees for his visits, and who at the same time makes a bargain with a druggist under which he charges his patient three shillings for the medicine which will cost him but two, can scarcely, we think, be said to maintain the dignity of a learned profession. Such an arrangement bears some sort of resemblance to what is described as a "secret commission," and, at the very least, it is one that ought to be disclosed to the patient, leaving him the option of having his prescriptions dispensed elsewhere. The case is rendered worse by what appears to have happened in the case under consideration, in which the patient not only obtained secret doses of chloral from the druggist in excess of those prescribed, but in which these secret doses were absolutely made to carry the profit of 50 per cent. to the plaintiff. The plaintiff will, of course, be put to heavy costs by his unsuccessful litigation; and it does not appear to us that he is in a position to derive comfort from the thought that he has defended a principle for which it was wise and right to contend.

